# Drug supply shortage in Nigeria during COVID-19: efforts and challenges

**DOI:** 10.1186/s40545-021-00302-1

**Published:** 2021-01-22

**Authors:** Edward Faiva, Hashim Talib Hashim, Mustafa Ahmed Ramadhan, Shingin Kovona Musa, John Bchara, Yahya Dheyaa Tuama, Yusuff Adebayo Adebisi, Mustafa Hayder Kadhim, Mohammad Yasir Essar, Shoaib Ahmad, Don Eliseo Lucero-Prisno

**Affiliations:** 1 Faculty of Pharmaceutical Sciences, Ahmadu Bello University, Zaria, Nigeria; 2 College of Medicine, University of Baghdad, Baghdad, Iraq; 3 Faculty of Medicine, Tishreen University, Lattakia, Syria; 4 Faculty of Pharmacy, University of Ibadan, Ibadan, Nigeria; 5 College of Medicine, University of Kufa, Kufa, Iraq; 6 Medical Research Center, Kateb University, Kabul, Afghanistan; 7 Kabul University of Medical Sciences, Kabul, Afghanistan; 8 Punjab Medical College, Faisalabad, Pakistan; 9 Department of Global Health and Development, London School of Hygiene and Tropical Medicine, London, UK; 10 Faculty of Management and Development Studies, University of the Philippines (Open University), Los Banos, Laguna Philippines

**Keywords:** COVID-19, Drug shortage, Nigeria, Efforts, Pharmaceutical

## Abstract

The COVID-19 pandemic has resulted in massive disruptions in global supply chains. Nigeria is particularly vulnerable with respect to pharmaceuticals since there is reduced local production and about 70% of the drug supply is imported creating a huge supply–demand disparity particularly in times like COVID-19. Nigeria is in need of huge quantities of quality-assured health commodities to effectively respond to the pandemic. Significant shortages of other essential medicines and medical products across the country could be imminent. Drug scarcity in Nigeria during the COVID-19 pandemic period is because of several accumulated factors, majorly as a result of global lockdown, decreased manufacturing, unaddressed regulatory affairs, poor access to resources by the population, lack of buffer stocks, security instability, and poor funding of the healthcare system. This situation if left unattended, could cause serious drawbacks to the health of the populace as well as the quality of life of Nigerians amid the COVID-19 Pandemic. Appropriate measures should be directed to ensure ethical processes on drug production, importation, pricing, and distribution to avoid such events during unavoidable scenarios, like the COVID-19 pandemic and other public health emergencies.

## Introduction

The leading causes of death and disability in Nigeria are preventable and treatable diseases that can be alleviated with cost-effective essential medicines. However, majority of the population lacks regular access to essential medicines. Many of those who are able to access it are either given the wrong treatment, receive too little medicine for their health conditions, or use the medicines incorrectly [1]. The effective management of drug supply addresses practical ways in which government policy-makers, essential medicines program managers, non-governmental organizations (NGOs), donors, and others can work to ensure that high-quality essential medicines are available, affordable and are rationally consumed. Medicines are of critical importance because they can save lives and improve health. Moreover, they promote trust and participation in health services. They can be expensive and with special concerns bring a uniqueness that sets them apart from other consumer products [2]. Many essential drugs are currently short in Nigeria with the supply of antiretroviral being the most threatened.

The Chairman of Pharmaceutical Manufacturers Group of the Manufacturers Association of Nigeria (PMG-MAN), in January 2019, made a case for drug insecurity in Nigeria. He urged the federal government to increase local production of essential drugs from the current 40 to 75%. Moreover, he demanded that at least 300 billion Naira should be injected into the sector to enable players in the pharmaceutical industry to ramp up local production rather than depending solely on import products. In line with this interest, and that of the Pharmaceutical Society of Nigeria (PSN), that have suggested policies that would encourage local manufacturing of medicines, the federal government jacked up the tax for importation of essential drugs up to 20% as part of measures to encourage pharmaceutical firms to manufacture medicines locally [2, 4].

In 1990, the maiden National Drug Policy for Nigerians launched to address the challenges associated with the inadequacies of drug supply and distribution These inadequacies resulted from various factors, such as: an ineffective structure of drug administration and control, inadequate funding of drug supply and control activities, increased reliance on foreign sources for finished drug products, pharmaceutical raw materials, reagents and equipment, inefficient storage, transportation and distribution facilities, poor selection and procurement practices, involvement of incompetent persons in procurement, distribution and sale of drugs, poor performance of drug suppliers to public health care institutions, and lack of political will to provide safe, efficacious and good quality drugs to meet the health needs of Nigerians. The policy was created with commendable goals and objectives intended to deal with the unsatisfactory situation at that time. The adoption of this policy was an encouraging development by the observers. After more than a decade of its adoption and implementation, modest progress has been reported. The positives to take are the publication of an Essential Drugs List and a National Drug Formulary, the establishment of a statutory agency responsible for drug administration and control, and the introduction of drug registration procedures.

Regardless, a lot remains to be done in many areas, including the actualization of self-sufficiency in local production of essential drugs, establishment of an effective drug procurement system, development of a well-ordered drug distribution system, harmonization and updating of drug legislation, effective control of drug advertisement and promotion, entrenchment of and commitment to rational drugs use at all levels of healthcare, and drug research and development, etc. [5, 6]. The revision of the policy presents an excellent opportunity for the formulation of new strategies, strengthening achievements in areas where progress has been recorded, and addressing those areas that call for more effective action. It is expected that with judicious implementation of the revised policy, as laid out in the accompanying implementation plan, the Nigerian people will have sustainable access to safe, efficacious and good quality drugs [6].

## Factors influencing drug shortage in Nigeria

In addition to the importation of finished products, wholesale and retail pharmacy businesses have been flourishing in Nigeria. There has also been a parallel growth in local drug manufacturing, coupled with a very high demand of industrial conditions and standards required for both raw materials, dosage form processing, equipment, and processing environment [7, 8]. Moreover, the control of global drug business by multinational cooperation that has overcome most of their initial development problems, has resulted in unfair competition from imported products and multinationals against the locally manufactured products [9].

The need to standardize many drugs of herbal origin circulating in Nigeria, and the inability of the countries to put to good use the research results from their basic or applied scientists has led to brain drain to foreign laboratories. There is still the inadequacy on the part of the government to check illegal importation, manufacture, and sale of fake, adulterated, substandard and expired goods due to fraudulent drug dealers and some corrupt government officials. Lack of effective research and development due to poor research support from the government and private companies has also been a major factor associated with drug scarcity in Nigeria [10, 11].

## Conclusion and recommendations

With the threat of the COVID-19 pandemic still looming, it is high time that the federal government removes bottlenecks on importation of drugs and devises other effective ways of ensuring that Nigeria is not at any time cut off from the supply of essential drugs.

There is dire need to achieve financial sustainability through greater efficiency and financing mechanisms that will increase availability while ensuring equity. Efficiency in public pharmaceutical supply through strategies that build on public-sector strengths should be ensured, while also incorporating greater flexibility and competitiveness. More effort should also be directed at changing the behavior of providers, patients, and the public to promote effective, safe, and economical prescribing, dispensing, and patient use of medicines. The role of government in improving the availability, affordability, and rational use of medicines in the private sector should also be strengthened. Through adoption and enforcement of legislations and regulations, the government should also regulate the safety, efficacy and quality of drugs and medical products as to ensure all medicines meet basic quality standards.

## Data Availability

Not applicable.

## Abbreviations

NGOs: Nongovernmental organizations
PMG-MAN: The Chairman of Pharmaceutical Manufacturers Group of the Manufacturers Association of Nigeria
PSN: Pharmaceutical Society of Nigeria
